# Antigen Peptide Transporter 1 (TAP1) Promotes Resistance to MEK Inhibitors in Pancreatic Cancers

**DOI:** 10.3390/ijms23137168

**Published:** 2022-06-28

**Authors:** Boya Li, Yu Feng, Qiaoyun Hou, Yan Fu, Yongzhang Luo

**Affiliations:** 1 Cancer Biology Laboratory, School of Life Sciences, Tsinghua University, Beijing 100084, China; liby15@mails.tsinghua.edu.cn (B.L.); feng-y19@mails.tsinghua.edu.cn (Y.F.); hqy16@mails.tsinghua.edu.cn (Q.H.); 2 Beijing Key Laboratory for Protein Therapeutics, Tsinghua University, Beijing 100084, China; 3 The National Engineering Research Center for Protein Technology, Tsinghua University, Beijing 100084, China

**Keywords:** TAP1, ABCB2, MEK inhibitor, drug resistance, PDAC

## Abstract

Mitogen-activated protein kinase (MAPK) kinase (MEK) inhibitors show limited benefit in Kirsten rat sarcoma (*KRAS*) mutant pancreatic cancer due to drug resistance. To identify mechanisms of resistance to MEK inhibitor (MEKi), we employed a differential expression analysis of MEKi-sensitive versus MEKi-resistant *KRAS*-mutant pancreatic cancer cell lines. Here, we report that the antigen peptide transporter 1 (TAP1) expression levels of MEKi-resistant cell lines were notably higher than those of MEKi-sensitive cell lines. Suppression of TAP1 significantly sensitized the MEKi-resistant pancreatic ductal adenocarcinoma (PDAC) cells to MEKi and induced higher apoptotic rate in vitro. Moreover, knockdown of TAP1 in MEKi-resistant tumor significantly decreased tumor growth in vivo. Consistently, overexpression of TAP1 in sensitive PDAC cells resulted in increased resistance to MEKi, both in vitro and in vivo. Mechanistic studies demonstrated that TAP1 promoted chemoresistance by enhancing the transport of MEKi out of PDAC cells, leading to reduced intracellular MEKi concentration and attenuated inhibition of KRAS signaling pathways. Moreover, TAP1 expression increased spheroid formation abilities of PDAC cells. These findings suggest that TAP1 could serve as a potential marker for predicting the response of patients to MEKi. Combination of TAP1 suppression and MEKi may provide a novel therapeutic strategy for PDAC treatment.

## 1. Introduction

Pancreatic cancer ranks as the seventh leading cause of cancer death worldwide in 2020. It is recognized as one of the most fatal cancers, with a mortality almost equal to the incidence (466,003 deaths vs. 495,773 new cases) [1]. The overall five-year survival rate of pancreatic cancer patients is around 10% [2]. The poor survival rates and treatment failure in pancreatic cancer are attributable to several factors, most notably chemoresistance [3].

Pancreatic ductal adenocarcinoma (PDAC) is the most common form of pancreatic cancer, representing more than 90% of pancreatic tumor. More than 95% of PDAC has an activating *KRAS* mutation, resulting in aberrant signaling through the mitogen-activated protein kinase (MAPK) pathway to promote tumor progression [4,5,6]. The most prevalent and well-studied mutation in *KRAS* is at codon 12, in which glycine mutates to aspartate (G12D) on most occasions, then to valine (G12V), cysteine (G12C), or arginine (G12R) by frequency [7]. Although specific inhibitors targeting the *KRAS* (G12C) mutant have recently been developed in solid tumors, most mutant forms of *KRAS* are still undruggable [8,9]. Being the downstream effector of KRAS, MAPK kinase (MEK) has been one of the most attractive targets for treating *KRAS*-mutant tumors [10,11]. Nevertheless, MEK inhibitors (MEKi) show limited efficacy in preclinical and clinical trials, often due to the occurrence of drug resistance [12,13,14]. Thus, comprehensive understanding of the molecular mechanisms underlying MEKi resistance in PDAC is an urgent need.

The ATP-binding cassette (ABC) transporters are known to play an important role in chemoresistance due to their function as drug efflux pump [15,16,17]. The ABC transporters family consists of 49 transporters in seven subfamilies (ABCA to ABCG), which regulate the intracellular content of various xenobiotics, peptides, and other small molecules [18]. The most extensively studied member related to drug efflux pump is ABCB1 (also named P-glycoprotein). It can transport various chemotherapeutic agents out of cells leading to multidrug resistance [19]. TAP1, also known as ABCB2, is a member of the ABCB subfamily. It is responsible for the pumping of degraded cytosolic peptides from cytosol to ER lumen [20,21]. A gene screening of 23 ABC transporters identified a marked increase in TAP1 expression in the gemcitabine-resistant variants of pancreatic cancer cells [22]. However, the essential function of TAP1 and its underlying mechanism in regulating the chemosensitivity of human cancer remain largely unknown.

In this study, we demonstrated that TAP1 enhanced the MEKi resistance of PDAC cells by transporting MEKi out of cells, which suggests that targeting TAP1 improves the efficacy of MEKi treatment in patients with *KRAS*-mutant PDAC.

## 2. Results

### 2.1. KRAS-Driven Pancreatic Cancer Cells Have Different Sensitivities to MEK Inhibition

**Figure 1:**
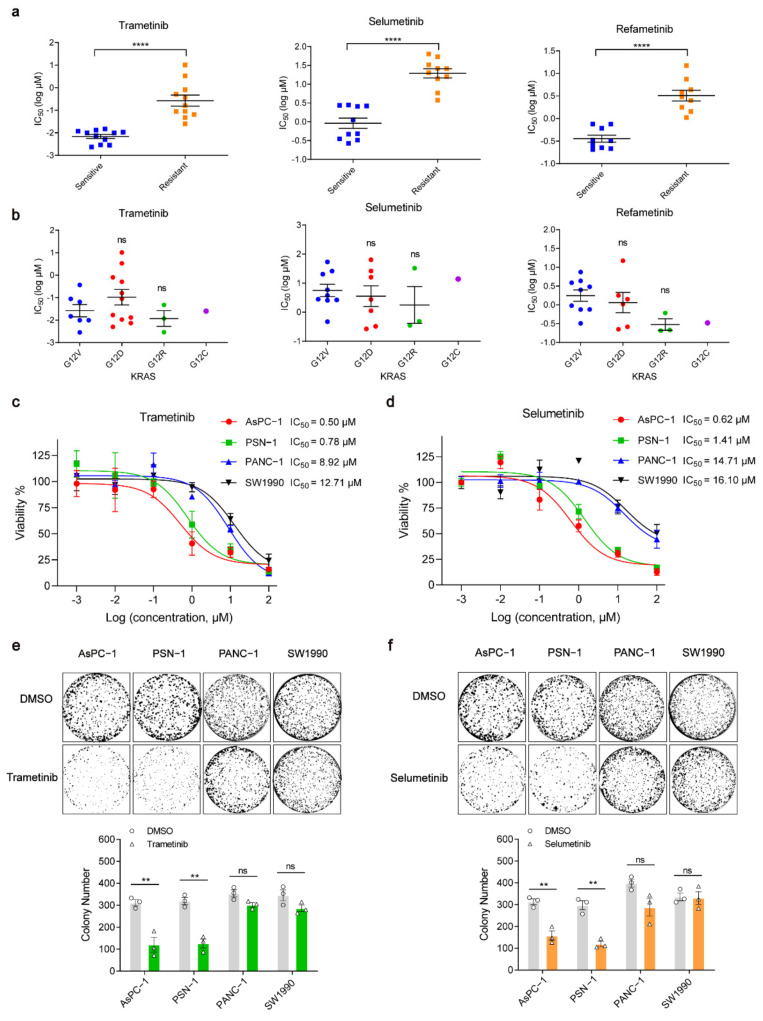
*KRAS*-driven pancreatic cancer cells have different sensitivities to MEK inhibition. (**a**) Comparison of the sensitivity of *KRAS*-mutant pancreatic cancer cell lines treated by different kinds of MEK inhibitors. **** *p* < 0.0001. Information of logIC_50_ was from the Genomics of Drug Sensitivity in Cancer (GDSC) database. (**b**) IC_50_ values of four subtypes of mutant amino acid at *KRAS* codon 12. Average IC_50_ values of cell lines with specific KRAS mutational subtypes were compared with average IC_50_ values of cell lines with *KRAS* (G12V) to determine the *p*-value, Mann–Whitney test, ns, not significant. (**c**,**d**) Cell viabilities of SW1990, PANC-1, AsPC-1, and PSN-1 cell lines treated for 72 h with trametinib or selumetinib. (**e**,**f**) Colony formation of the cell lines in (**c**,**d**) were assessed for 12 days with DMSO, trametinib (100 nM) or selumetinib (200 nM), respectively. ** *p* < 0.01; ns, not significant. Two-tailed t test. Representative results from at least three of the biological replicates are shown per condition.

Given only some patients benefit from MEK inhibitors in the treatment of *KRAS*-mutant pancreatic cancers, we were encouraged to investigate whether different *KRAS*-mutant human pancreatic cancer cell lines respond differently to MEK inhibition. Five most commonly used MEKis (trametinib, selumetinib, refametinib, CI-1040, and PD0325901) were recommended for further study by GDSC database (Genomics of Drug Sensitivity in Cancer). About 20 kinds of *KRAS*-mutant pancreatic cancer cell lines (listed in Supplementary Material, Table S1) were defined into either sensitive or resistant group, according to the median half-maximal inhibitory concentration (IC_50_) values of each MEKi in these cell lines. IC_50_ values of resistant group were significantly higher than those of sensitive group in all the five MEKis mentioned above (Figure 1a and Supplementary Figure S1a). Considering the heterogeneity of mutation subtypes in *KRAS* [23], *KRAS*-mutant pancreatic cancer cell lines were grouped by their mutational status at codons 12 (G12V, G12D, G12R, and G12C). However, no statistical significance was observed in IC_50_ values of each MEKi among these mutational subtypes (Figure 1b and Supplementary Figure S1b). It was indicated that there was another mechanism independently of *KRAS* mutation subtypes to explain the different responses of pancreatic cancer cells to MEKi.

To further validate these results obtained from the database, we selected two MEKi-resistant cell lines (SW1990 and PANC-1) and two MEKi-sensitive cell lines (AsPC-1 and PSN-1) to perform cell viability and colony formation assays. The PANC-1 and SW1990 cells were more resistant to trametinib, represented by a 15.90-fold increase in the average IC_50_ (10.81 μM), as compared with AsPC-1 and PSN-1 cells (the average IC_50_: 0.64 μM; Figure 1c). A similar result was observed with selumetinib treatment; the average IC_50_ values of each of the above two cell groups were 15.40 μM and 1.01 μM, respectively (Figure 1d). In colony formation assays, trametinib and selumetinib showed significant inhibitory effect on colony numbers of AsPC-1 and PSN-1 cells, whereas there was no remarkable reduction in PANC-1 and SW1990 cells (Figure 1e,f). In addition, both AsPC-1 and SW1990 cells are with *KRAS* G12D mutation, while they had a huge difference in IC_50_ values of trametinib and selumetinib. It was necessary to explore the molecular mechanism independently of *KRAS* mutation subtypes in MEKi-sensitive and -resistant cell lines.

### 2.2. KRAS-Mutant PDAC Cell Lines with Higher Expression of TAP1 Are More Resistant to MEKi

**Figure 2:**
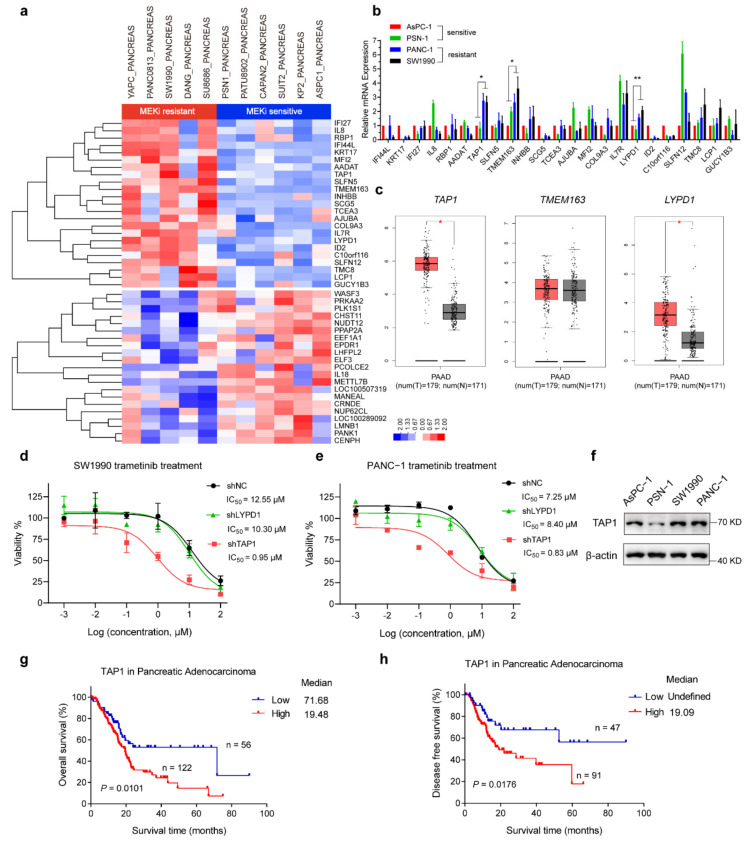
*KRAS*-mutant PDAC cell lines with high TAP1 are resistant to MEK inhibition. (**a**) Unsupervised hierarchical clustering of 11 *KRAS*-mutant pancreatic cancer cell lines from the CCLE database. Log2 transformed fold change expression levels are indicated by the color gradient. (**b**) The mRNA levels of 23 upregulation genes assessed by qRT-PCR in MEKi-resistant and -sensitive cell lines. * *p* < 0.05; ** *p* < 0.01; and one-way ANOVA test with Tukey’s multiple comparisons. (**c**) The mRNA expression levels of *TAP1*, *TMEM163*, and *LYPD1* on box plots from GEPIA. Red color means tumor samples, n = 179; gray color means normal tissues, n = 171. One-way ANOVA analysis, * *p* < 0.05. (**d**,**e**) Trametinib sensitivity of SW1990 or PANC-1 cells with shTAP1 and shLYPD1 were determined by CellTiter-Lumi™ assays. Nontargeting shRNA (NC) was used as control (**f**) Western blot analysis of TAP1 expression in AsPC-1, PSN-1, SW1990, and PANC-1 cell lines. (**g**,**h**) Kaplan–Meier curve for overall survival and disease-free survival of the pancreatic adenocarcinoma patients based on the mRNA expression level of TAP1 from TCGA dataset.

To investigate the potential cause for these significant alterations in the sensitivity to MEKi, we carried out unsupervised hierarchical clustering of 11 *KRAS*-mutant pancreatic cancer cell lines from the Cancer Cell Line Encyclopedia (CCLE). Five of the 11 cell lines were categorized as MEKi-resistant group and the other six cell lines as MEKi-sensitive according to their IC_50_ values from the GDSC database. A total of 44 differential expression genes (23 upregulated, 21 downregulated) were selected in the MEKi-resistant group, compared to MEKi-sensitive groups (Figure 2a). The 23 upregulated genes were subjected to further validate their mRNA expression levels in four PDAC cell lines (SW1990, PANC-1, AsPC-1, and PSN-1). The mRNA expression levels of three genes (TAP1, TMEM163, and LYPD1) were significantly higher in the MEKi-resistant cells (SW1990 and PANC-1), compared to those in the MEKi-sensitive cells (AsPC-1 and PSN-1) (Figure 2b).

Next, we used Gene Expression Profiling Interactive Analysis (GEPIA) to analyze their mRNA expression level in normal and pancreatic tumor tissues. The mRNA levels of *TAP1* and *LYPD1* in tumor tissues were significantly higher than those in normal tissues, whereas no significant difference was observed in the mRNA levels of *TMEM163* (Figure 2c). To assess the function of TAP1 and LYPD1 in MEKi resistance, we knocked down TAP1 and LYPD1 in SW1990 and PANC-1 cell lines (Supplementary Figure S2a,b). Interestingly, suppression of TAP1 dramatically decreased the IC50 values of trametinib, both in SW1990 cells (from 12.55 μM to 0.95 μM; Figure 2d) and PANC-1 cells (from 7.25 μM to 0.83 μM; Figure 2e). Consistently, the IC_50_ values of selumetinib was also largely reduced both in SW1990 and PANC-1 cells by TAP1 knockdown (Supplementary Figure S2c,d). However, LYPD1 knockdown failed to improve the sensitivity of SW1990 or PANC-1 cells to MEKis (Figure 2d,e and Supplementary Figure S2c,d). In addition, knockdown of TAP1 did not affect the baseline proliferation of SW1990 or PANC-1 cells, as suggested by cell counting (Supplementary Figure S2e,f). Higher protein levels of TAP1 in SW1990 and PANC-1 cells were observed, compared to those in AsPC-1 and PSN-1 cells (Figure 2f). These results revealed that increased TAP1 expression was associated with MEKi resistance in PDAC.

To investigate the clinical relevance of TAP1 in human pancreatic cancer patients, we analyzed the expression of TAP1 in pancreatic adenocarcinoma cohort from TCGA. Patients with low expression levels of TAP1 (z-score < −0.5) showed better overall survival rate (Figure 2g) and disease-free survival rate (Figure 2h) than patients with high expression levels of TAP1 (z-score > −0.5), suggesting that TAP1 exerted a cancer-promoting role in pancreatic adenocarcinoma.

### 2.3. TAP1 Suppression Sensitizes the MEKi-Resistant PDAC Cells to MEKi Treatment

**Figure 3:**
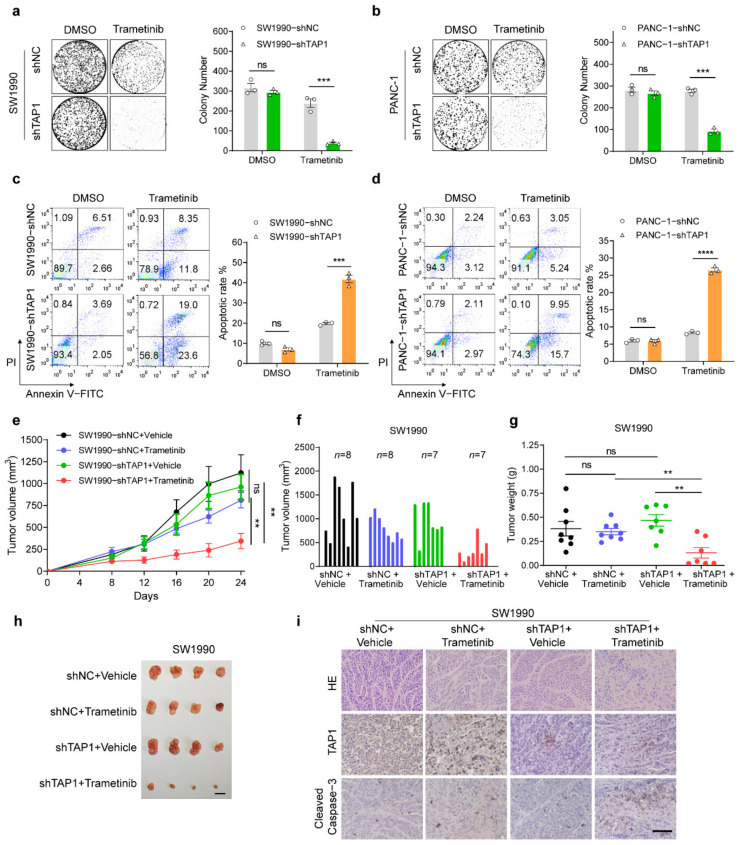
Suppression of TAP1 sensitizes the MEKi-resistant PDAC cells in vitro and in vivo. (**a**,**b**) Representative wells of the clonogenic growth assay upon knockdown of TAP1 or NC in SW1990 and PANC-1 cells after trametinib (100 nM) treatment for 12 days. Quantification of colony number is shown at the right. (**c**,**d**) Apoptosis rates of SW1990 and PANC-1 cells with shTAP1 or shNC were detected by Annexin V-FITC/PI detection kit after DMSO or trametinib (40 μM) treatment for 72 h. The quantitative results are shown at the right. (**e**) Tumor volume measurements at the indicated time points for SW1990-shNC and SW1990-shTAP1 tumors, treated with trametinib (1 mg/kg) or vehicle control by via oral gavage daily. (**f**,**g**) Tumor volume and tumor mass at the endpoint of experiments. (**h**) Images of four representative primary subcutaneous tumors for each of the indicated treatment groups. Scale bar, 1 cm. (**i**) Hematoxylin and eosin (H and E) staining and IHC staining of TAP1 or cleaved caspase-3 in primary tumor tissues. Scale bars, 200 μm. Two-tailed *t* test. ** *p* < 0.01; *** *p* < 0.001; **** *p* < 0.0001; and ns, not significant.

Since TAP1 knockdown reduced the IC50 values of MEKi for SW1990 and PANC-1 cells, we further verified the promoting role of TAP1 in cell proliferation by colony formation assays. Colony numbers of SW1990 and PANC-1 cells were dramatically reduced by TAP1 knockdown under trametinib treatment (Figure 3a,b). Moreover, knockdown of TAP1 in SW1990 and PANC-1 cells significantly promoted the apoptotic rates caused by trametinib (Figure 3c,d), compared to their control (shNC) group. Similar results were acquired in SW1990 and PANC-1 cells by selumetinib treatment (Supplementary Figure S3a–d). It was demonstrated that TAP1 suppression enhanced the inhibitory effect on proliferation and the promotion effect on apoptosis of MEKi in MEKi-resistant cells.

Subsequently, the effect of TAP1 suppression on the chemo-response of MEKi-resistant PDAC was examined in vivo. Xenograft model was constructed by inoculating SW1990-shNC and SW1990-shTAP1 cells subcutaneously into nude mice following treatment with trametinib or vehicle. In SW1990-shNC xenografts, trametinib resulted in a modest, but not significant, 27.8% reduction in the average final tumor volume (810.8 ± 206.3 mm^3^), compared to the vehicle-treated control, with an average final tumor volume of 1123 ± 206.3 mm^3^. Whereas in SW1990-shTAP1 xenografts, trametinib resulted in an average final tumor volume of 344.0 ± 86.1 mm^3^, representing a significant 64.4% reduction (*p* = 0.0031) in tumor volume compared to vehicle-treated tumors (average final tumor volume: 966.4 ± 143.8 mm^3^; Figure 3e,f). The consistent results were acquired in tumor weights (Figure 3g,h). Additionally, trametinib showed little effect on the expression level of TAP1 in both SW1990-shNC and SW1990-shTAP1 xenografts, as detected by IHC analysis (Figure 3i and Supplementary Figure S3e). The expression levels of cleaved caspase-3 in SW1990-shTAP1 xenografts were significantly higher than those in SW1990-shNC xenografts under trametinib treatment (Figure 3i; Supplementary Figure S3f). These studies demonstrated that TAP1 suppression dramatically inhibited the MEKi resistance in PDAC, which resulted in reduced tumor growth and increased levels of cleaved caspase-3.

### 2.4. Ove-rexpression of TAP1 Renders PDAC Cells More Resistant to MEKi

**Figure 4:**
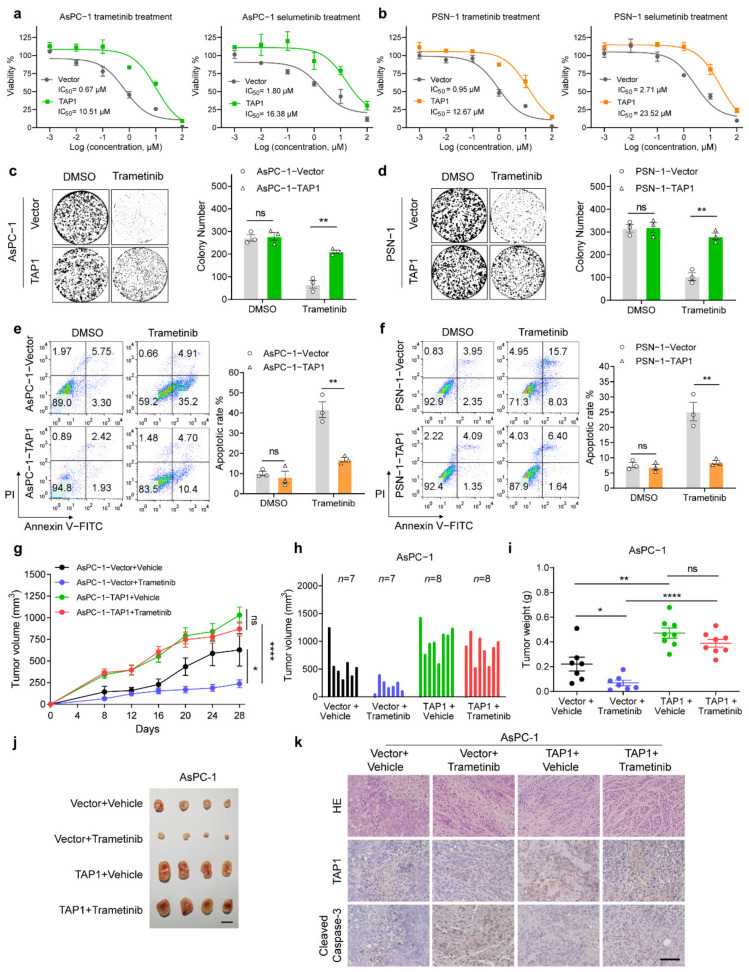
Overexpression of TAP1 renders PDAC cells more resistant to MEKi in vitro and in vivo. (**a**) Trametinib and selumetinib sensitivities of AsPC-1 cells with TAP1 overexpression compared with control (Vector) cells. (**b**) Trametinib and selumetinib sensitivities of PSN-1 cells with TAP1 and vector control. (**c**,**d**) Representative wells of colony formation assay in TAP1-overexpressing AsPC-1 and PSN-1 cells compared with their vector control cells, treated by trametinib (100 nM) for 12 days. Quantification of colony number is shown at the right. (**e**,**f**) Apoptotic rates of TAP1-overexpressing cells and their vector control cells were detected by Annexin V-FITC/PI detection kit after trametinib (40 μM) treatment for 72 h. The quantitative results are showed at the right. (**g**) Tumor volume measurements at the indicated time points for AsPC-1-RFP and AsPC-1-TAP1 tumors, treated with trametinib (1mg/kg) or vehicle control by via oral gavage daily. (**h**,**i**) Tumor volume and tumor weights at the endpoint of experiments. (**j**) Images of four representative primary subcutaneous tumors for each of the indicated treatment groups. Scale bar, 1 cm. (**k**) H and E staining and IHC staining of TAP1 or cleaved caspase-3 in primary tumor tissues at the endpoint of experiments. Scale bars, 200 μm. Two-tailed *t* test. * *p* < 0.05; ** *p* < 0.01; **** *p* < 0.0001; and ns, not significant.

To further determine the function of TAP1 in MEKi resistance, we constructed the TAP1 overexpression cell lines in AsPC-1 and PSN-1 cells (Supplementary Figure S4a). Overexpression of TAP1 in AsPC-1 cells significantly increased the IC_50_ value of trametinib from 0.67 μM to 10.51 μM, and the IC_50_ value of selumetinib was also notably increased from 1.80 μM to 16.38 μM (Figure 4a). Meanwhile, the IC_50_ values of trametinib and selumetinib were also remarkably increased by TAP1 overexpression in PSN-1 cells (Figure 4b). However, TAP1 overexpression did not improve the baseline proliferation of AsPC-1 or PSN-1 cells (Supplementary Figure S4b). In colony formation assay, the number of resistant colonies in AsPC-1 and PSN-1 cells was significantly increased by TAP1 overexpression after trametinib (Figure 4c,d) and selumetinib treatment (Supplementary Figure S4c,d). Overexpression of TAP1 also significantly inhibited the apoptotic rate of AsPC-1 and PSN-1 cells induced by trametinib (Figure 4e,f) and selumetinib (Supplementary Figure S4e,f). These results indicated that TAP1 overexpression promoted MEKi resistance in PDAC cell lines.

To evaluate the effect of TAP1 overexpression on MEKi-response in vivo, AsPC-1-Vector and AsPC-1-TAP1 cells were used to construct xenograft models. In AsPC-1-Vector xenografts, trametinib resulted in a significant 64.2% decreased in the average final tumor volumes (211.2 ± 43.64 mm^3^, *p* = 0.0105), compared to the vehicle-treated control (average final tumor volume: 589.5 ± 117.2 mm^3^). However, in AsPC-1-TAP1 xenografts, trametinib resulted in an average final tumor volume of 870.5 ± 81.57 mm^3^, showing a slight 15.6% decreased in tumor volume compared to vehicle-treated tumors (average final tumor volume: 1031 ± 92.88 mm^3^; Figure 4g,h). Consistent results were obtained in tumor weights (Figure 4i,j). IHC staining of tumor samples showed that the expression of TAP1 was not influenced by trametinib (Figure 4k and Supplementary Figure S4g). Expression levels of cleaved caspase-3 induced by trametinib were dramatically decreased by TAP1 overexpression in AsPC-1 tumors (Figure 4k and Supplementary Figure S4h). These results demonstrated that TAP1 overexpression rendered PDAC xenografts more resistant to MEKi treatment.

### 2.5. TAP1 (ABCB2) Expression Promotes the Efflux of MEKi and Attenuates Inhibitory Effects in PDAC Cells

The chemoresistance of cancer cells is related to the high expression of multiple ABC transporters, which locate in the membrane with the function of drug efflux [24]. TAP1, also known as ABCB2, has been reported to localize in the endoplasmic reticulum (ER) membrane and transport peptide antigens from cytosol to ER [25]. In view of the functional redundancy of the highly conserved ABC transporters family [26], it is reasonable to expect the function of TAP1 (ABCB2) in drug efflux. The increased TAP1 expression on the cell surface was observed in AsPC-1 and PSN-1 cells by TAP1 overexpression (Supplementary Figure S5a,b). Knockdown of TAP1 significantly reduced its expression on the cell surface of SW1990 and PANC-1 cells (Supplementary Figure S5c,d). It was indicated that the membrane-associated TAP1 participated in drug efflux.

**Figure 5:**
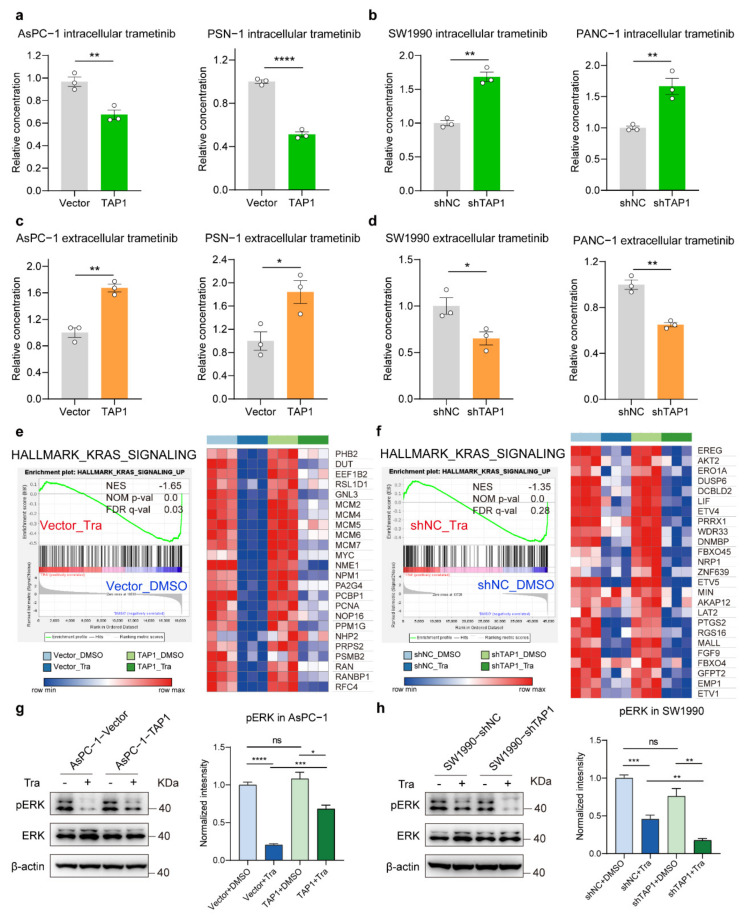
TAP1 (ABCB2) expression promotes the efflux of MEKi and attenuates inhibitory effects in PDAC cells. (**a**) Intracellular drug accumulation after incubation with 1 μM trametinib in TAP1-overexpressing and their vector control cells were determined by LC-MS/MS. (**b**) Detection of drug accumulation in TAP1-knockdown and their control cells treated by 1 μM trametinib. (**c**,**d**) Measurement of extracellular trametinib in the cell culture medium of TAP1-overexpressing cells and TAP1-knockdown cells by LC-MS/MS, compared to their respective vector control cells. (**e**,**f**) Gene set enrichment analysis (GSEA) of genes significantly downregulated in AsPC-1-Vector and SW1990-shNC cells treated by trametinib in comparison with DMSO treatment. The normalized enrichment score (NES), the nominal (NOM) *p*-value, and the false discovery rate (FDR) q-value are indicated in the insert. At the right, each panel shows a heat map with the core genes of each gene set in different groups. (**g**,**h**) The expression level of phosphorylated ERK (pERK) in TAP1-overexpressing (AsPC-1-TAP1) and TAP1-knockdown (SW1990-shTAP1) cells, compared to their respective control cells, treated by trametinib (100 nM) or DMSO for 72 h. Mean ± SEM of results was shown. Two-tailed *t* test; * *p* < 0.05; ** *p* < 0.01; *** *p* < 0.001; **** *p* < 0.0001; and ns, not significant.

Therefore, we tested the intracellular MEKi accumulation in PDAC cells by LC-MS/MS analysis system. TAP1 overexpression significantly reduced the intracellular concentration of trametinib in AsPC-1 and PSN-1 cells, compared to their respective control (Figure 5a). Similar results were obtained in these cells treated by selumetinib (Supplementary Figure S5e). Moreover, knockdown of TAP1 significantly increased the intracellular concentrations of MEKis both in SW1990 and PANC-1 cells (Figure 5b and Supplementary Figure S5f). These results led us to propose that the decreased intracellular MEKi was attributed to the function of TAP1 in drug efflux. Thus, we examined the concentration of MEKis in the cell culture supernatant. Overexpression of TAP1 significantly increased the concentrations of trametinib and selumetinib in cell culture medium of TAP1-overexpressing cells, compared to their vector control cells (Figure 5c and Supplementary Figure S5g). Consistently, the extracellular concentrations of trametinib and selumetinib were dramatically decreased in SW1990 and PANC-1 cells by TAP1 suppression (Figure 5d and Supplementary Figure S5h). These results suggested that TAP1 enhanced the efflux of MEKi out of PDAC cells, leading to a decreased intracellular drug accumulation.

MEK inhibitors were reported to inhibit ERK activation and its downstream processes leading to inhibitions of the proliferation of tumor cells [27]. To explore the effect of TAP1 on the signaling pathway influenced by MEKi, RNA-Seq analysis was performed both in AsPC-1 and SW1990 cells group. The gene set enrichment analysis (GSEA) in AsPC-1-Vector cells showed that hallmark gene sets related to KRAS were significantly downregulated by trametinib treatment, compared to DMSO treatment. The heatmap presented that AsPC-1-TAP1 cells had higher expression levels of this gene set than AsPC-1-Vector cells after trametinib treatment, indicating that TAP1 overexpression alleviated the inhibition of KRAS signaling pathway by trametinib (Figure 5e). Subsequently, the same analysis was performed in SW1990 cells group, showing that TAP1 knockdown enhanced the inhibitory effect of MEKi on KRAS signaling (Figure 5f). To further confirm these effects, the levels of phosphorylated ERK (pERK) in AsPC-1 and SW1990 cell lines treated by trametinib were detected. Trametinib treatment resulted in a robust decrease in pERK level in AsPC-1-Vector cells and a relatively weaker decrease in AsPC-1-TAP1 cells (Figure 5g). Additionally, trametinib resulted in a moderate inhibition on the level of pERK in SW1990-shNC cells and a much more profound inhibition in SW1990-shTAP1 cells (Figure 5h). These results demonstrated that TAP1 overexpression attenuated ERK signaling inhibition induced by trametinib, whereas TAP1 knockdown intensified this inhibition.

### 2.6. TAP1 (ABCB2) Expression Enhance the Stemness of PDAC Cells

**Figure 6:**
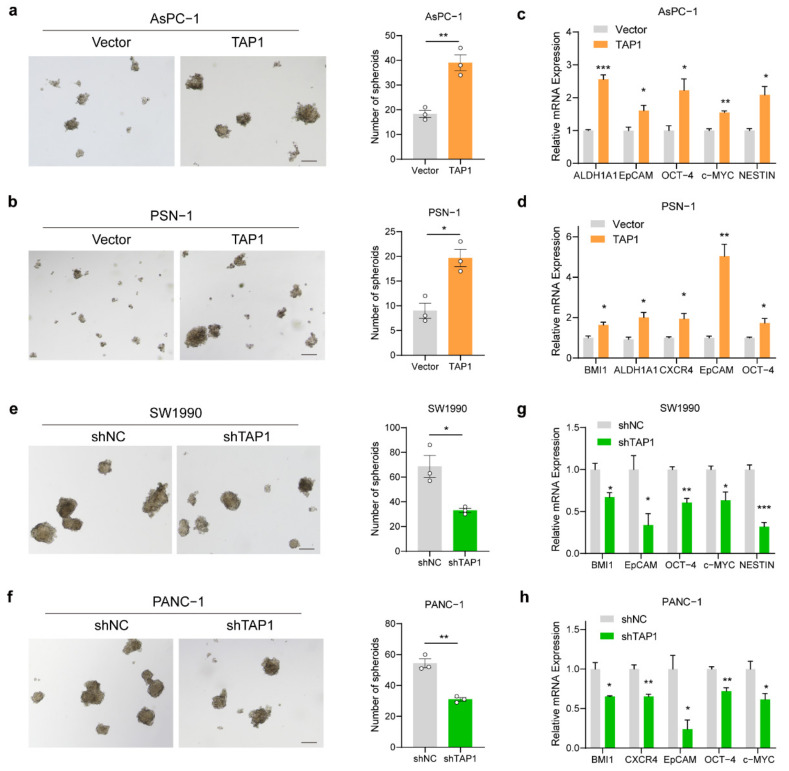
TAP1 (ABCB2) expression enhanced the stemness of PDAC cells. (**a**,**b**) Representative images of spheroid formation assay in TAP1-overexpressing cells compared with their vector control cells, cultured for 10 days. Scale bars 200 μm. Quantification of spheroids number is shown at the right. (**c**,**d**) The mRNA levels of cancer stemness-related markers genes assessed by qRT-PCR in TAP1-overexpressing and control (vector) cells. (**e**,**f**) Representative images of spheroid formation assay in TAP1-knockdown cells compared with their vector control cells, cultured for 10 days. Scale bar 200 μm. Quantification of spheroids number is shown at the right. (**g**,**h**) Detected by RT-qPCR. The reduction of BMI1, CXCR4, EpCAM, OCT-4, c-MYC, and NESTIN in TAP1-knockdown and control (shNC) cells was demonstrated. Mean ± SEM of results was shown. Two-tailed *t* test; * *p* < 0.05; ** *p* < 0.01; and *** *p* < 0.001.

It has reported that ABC transporters can promote cancer cell survival independently of drug efflux [20]. Given TAP1 expression increased tumor growth in vivo and overexpression of ABC transporters are predominantly found in cancer stem cells (CSCs) [28], we supposed that TAP1 expression could promote tumor stemness. To verify this hypothesis, we performed spheroid formation assay in TAP1-overexpresing and TAP1-knockdown cell models. In AsPC-1 and PSN-1 cell models, the number of spheroids was significantly increased by TAP1 overexpression (Figure 6a,b). BMI1, ALDH1A1, CXCR4, EpCAM, OCT-4, c-MYC, and NESTIN are cancer stemness-related markers of pancreatic cancer [29]. The mRNA expression levels of these markers were significantly upregulated by TAP1 overexpression in AsPC-1 and PSN-1 cells (Figure 6c,d). Furthermore, in SW1990 and PANC-1 cell models, TAP1-knockdown cells exhibited reduced spheroids-forming ability, compared to their control cells (Figure 6e,f). Knockdown of TAP1 significantly decreased the mRNA expression levels of stemness-associated factors (Figure 6g,h). Together, our results suggest that TAP1 expression enhanced the stemness of pancreatic cancer cells.

## 3. Discussion

One of the most frequently mutated oncogenes in human cancers is *KRAS*, which has been considered an undruggable target for decades. Targeting MEK is still an attractive therapy in the treatment of patients with *KRAS*-mutant cancers. Unfortunately, the response to MEKi is variable due to drug resistance. This emphasizes the need to uncover the underlying resistance mechanisms. In the present study, we identified that MEKi-resistant PDAC cells had notably higher expression of TAP1 than MEKi-sensitive cells. Knockdown of TAP1 sensitized resistant cells to MEKi, both in vitro and in vivo, indicated that TAP1 may serve as a potential marker to predict the sensitivity of patients to MEKi in clinical applications.

Our mechanism research showed that TAP1 enhanced the efflux of MEKi from PDAC cells, leading to decreased intracellular drug accumulation and increased tolerance to MEKi. TAP1 has been reported as a transporter associated with antigen processing, belonging to the ABCB (ATP binding cassette subfamily B) family. It plays an essential role in peptide delivery from cytosol to ER for the assembly of major histocompatibility complex (MHC) class I [30]. Previous studies have demonstrated that Hedgehog signaling enhances drug resistance by regulating the expression of TAP1 in PDAC and hepatocellular carcinoma [22,31]. However, the mechanism of TAP1 promoting drug resistance remains obscure. It has been discovered that TAP1 (ABCB2) and P-gp (ABCB1) share a high degree of homology in their transmembrane domains, which are thought to be the primary determinants of substrate specificity [32]. In our study, we discovered that TAP1 increased the efflux of trametinib and selumetinib out of PDAC cells. It was suggested that TAP1 could serve as a novel transporter of MEKi, but the precise mechanism of TAP1 in transporting MEKi remains to be discovered in future studies.

Although ABC transporters can pump cytotoxic drugs out of cancer cells and cause drug resistance, emerging evidence suggests that they have potential roles as active players in tumorigenesis and stemness [20,33]. In the mouse xenograft model, the tumor size and weight were significantly increased by the overexpression of TAP1 in AsPC-1 xenograft, compared to the control groups. This discrepancy could be explained by other influences of TAP1 on tumor development independently of drug efflux. In our study, we observed that TAP1 expression significantly improved spheroids-formation ability. Spheroid formation has been regarded as a representative trait of stem cells [34]. In a recent study, pathway and gene ontology (GO) features correlated to the TAP1 gene were analyzed in breast, lung, liver, and ovarian cancer, in which the cytokine–cytokine receptor pathway and chemokine signaling pathway are the most significant pathways influenced by TAP1 [35]. This study suggests that TAP1 may affect these pathways in PDAC and then remodel the tumor environment to promote tumor growth. The more mechanistic explanations for the functions of TAP1 independently of transporter in tumor progress remain to be established.

Resistance to MEK inhibition has long been investigated. Resistance mechanisms can be classified as intrinsic, adaptive, and acquired. There is no clear separation between these three mechanisms, since the same event can occur at different stages of treatment resistance [36]. The protein-tyrosine phosphatase SHP2 represents a common node downstream of RTKs that is required for RAS activation. Several independent studies have reported that SHP2 inhibitors prevent adaptive resistance to MEK inhibitors in RAS-driven cancers [37,38,39,40]. In our study, we explored a new resistance mechanism from the perspective of intrinsic resistance. TAP1 expression attenuated the response to MEKi by drug efflux. Additionally, the efflux is a ubiquitous mechanism responsible for both innate and acquired drug resistance [41]. Our previous data showed that TAP1 suppression decreased the reactivation of ERK phosphorylation, which was consistent with SHP2 inhibitors which blocked the adaptive increase in ERK phosphorylation [37]. These findings suggested that TAP1 also play a role in adaptive resistance of MEKi, which requires further investigation.

In conclusion, our study demonstrates that TAP1(ABCB2) enhances the MEKi resistance in PDAC cells through transporting MEKi out of cells and promotes cancer stemness. TAP1 may act as a predictor for the response of PDAC patients to MEKi and a promising therapeutic target for improving the efficacy of MEKi in the treatment of MEKi-resistant PDAC.

## 4. Materials and Methods

### 4.1. Cell Lines and Cell Cultures

SW1990, AsPC-1, PANC-1, and PSN-1 cell lines were obtained from the American Type Culture Collection (Manassas, VA, USA). 293T cell lines were from the China Infrastructure of Cell Line Resources (Beijing, China). All cell lines were authenticated using Short Tandem Repeat (STR) analysis, as described in 2012 in ANSI Standard (ASN-0002) by the ATCC Standards Development Organization (SDO) and in Capes-Davis and colleagues’ work [42]. All cell lines are routinely tested for mycoplasma using Myco-blue Mycoplasma Detector (D101-01, Vazyme, Nanjing, China). All experiments with these cell lines and their derivatives were conducted within six months of receipt or resuscitation after cryopreservation. All cells were cultured in DMEM supplemented with 10% FBS (Gibco BRL, Grand Island, NY, USA) plus 1% antibiotics (100 U/mL penicillin and 100 mg/mL streptomycin, HyClone Thermo scientific, Waltham, MA, USA). All cells were maintained in a 37 °C humidified incubator containing 5% CO_2_.

### 4.2. Antibodies and Chemical Reagents

Antibodies against Erk1/2 (4695), p-Erk1/2 (4377), TAP1 (12341, used for western blot), caspase-3 (9662), and cleaved caspase-3 (9661) were from Cell Signaling Technology (Danvers, MA, USA). Antibody of β-actin (ab8227) was from Abcam (Cambridge, UK). Antibody against TAP1 (11114-1-AP, used for immunohistochemistry and immunofluorescence) was from Proteintech (Chicago, USA). Antibody of LYPD1 (AP9745b) was from Abgent (San Diego, USA). MEK inhibitors trametinib (GSK1120212, S2673) were from Selleck (Houston, TX, USA). Selumetinib (AZD6244, T6218) were purchased from TargetMol (Boston, MA, USA). All reagents were stored according to the manufacturer’s instructions.

### 4.3. Cell Viability Assay

To determine the half-inhibitory concentration (IC50), 200 cells were seeded in 96-well plates and treated with concentrations of trametinib or selumetinib from 1 nM to 100 µM. After 72 h of treatment, cell viability was determined with the CellTiter-Lumi™ Luminescent Cell Viability Assay Kit (C0065L, Beyotime, Shanghai, China) by measuring the luminescent signal based on quantitation of the ATP present, according to the manufacturer’s instructions.

### 4.4. Colony Formation Assay

Cells were seeded into six-well plates in triplicates at a density of 2000 cells per well and cultured overnight. Then the medium of each well was replaced to fresh medium with drugs or vehicle every three days. After incubation for 10–15 days, colonies were fixed in 4% paraformaldehyde and stained with crystal violet. The number of individual colonies larger than 100 μm in diameter was counted using an inverted microscope (IX71; Olympus, Tokyo, Japan).

### 4.5. Quantitative Real-Time PCR

Total RNA was extracted using TRIzol Reagent (Invitrogen, Pittsburgh, PA, USA), then converted to cDNAs using Revert Aid First Strand cDNA Synthesis Kit (Thermo Fisher Scientific, Waltham, USA). qRT-PCR was performed using the SYBR Green qRT-PCR Master Mix Kit (Stratagene, San Diego, CA, USA), and the expressions of all used genes were measured by Mx3000P system. The results were normalized by β-actin and the control group. All primers in this study were synthesized by RuiBiotech (Beijing, China) and were listed in Supplementary Material, Table S2.

### 4.6. Lentivirus Production and Infection

The lentivector expression plasmids, the packaging vector psPAX2, the envelope plasmid pVSVG, and the transfer plasmid SGEP containing the short hairpin RNA (shRNA) species targeting sequences for TAP1 mRNA (5′CCGGCCGTGTGTACTTATCCTGGATCTCGAGATCCAGGATAAGTACACACGGTTTTTG3′) and the sequences for LYPD1 mRNA (5′CCGGCCAG-GGAAACTGAACTCAGTTCTCGAGAACTGAGTTCAGTTTCCCTGGTTTTTTG3′), were transfected into cells using PEI reagent (Polysciences Inc., Warrington, UK). For stable overexpression lentivirus production, plasmid pLentiCMV was used as transfer plasmid. The viral supernatant was collected 48 h after transfection and filtered with 0.45 mm filter.

### 4.7. Cell Apoptosis Assay

Cells were seeded into six-well plates and the rates of apoptosis were measured by an Annexin V-FITC/PI Apoptosis Detection Kit (CWbiotech, Beijing, China) according to the manufacturer’s instructions. The flow cytometry analysis of apoptosis was performed using BD FACS Calibur (BD Biosciences, San Jose, CA, USA).

### 4.8. Spheroid Formation Assay

Spheroid formation assay was performed essentially as previously described [43]. For spheroid generation we used 20% of the methylcellulose stock solution (M0512, Sigma-Aldrich, St. Louis, MO, USA) and 80% culture medium corresponding to final 0.24% methylcellulose. The culture medium for all cells were used by DMEM-F12 supplemented with 10% FBS (F8318, Sigma-Aldrich, St. Louis, MO, USA). Low attachment 24-well plates (3473, Corning, NY, USA) at a density of 5000 cells/well. After 10 days of culture, the spheroids with diameter over 200 μm were counted and photographed.

### 4.9. Drug Accumulation and Efflux

To detect the intracellular accumulation of MEK inhibitors, 2 × 105 cells were seeded in 12-well plates and treated with 1 μM trametinib or 2 μM selumetinib for 4 h. The intracellular MEKi concentrations were measured by LC-MS/MS. To detect the extracellular concentration of MEK inhibitors, cells were washed twice with 1ml Hank’s solution to remove drug completely, after cells were treated with MEK inhibitors for 4 h. Then cells were added with 400 μL of blank serum-free mediums incubating for another 0.5 h at 37 °C. Concentrations of MEKi in supernatants were detected by LC-MS/MS. The LC-MS/MS system was a 6500plus QTrap mass spectrometer (AB SCIEX, Framingham, MA, USA) coupled with ACQUITY UPLC H-Class system (Waters, Milford, MA, USA). Multiple reaction monitoring (MRM) was performed at m/z values of 615.9/491.0 and 458.9/396.9 for trametinib and selumetinib, respectively.

### 4.10. Xenograft Studies

All animal studies were approved by the Institutional Animal Care and Use Committees of Tsinghua University (project number: 16-LYZ4). Different constructed pancreatic cancer cells (5 × 106, 1:1 in Matrigel, BD Biosciences) were inoculated subcutaneously into the right flank of 6–8 weeks old male BALB/c nude mice (Vital River, Beijing, China). Tumor volumes were measured every 3 or 4 days and were calculated by the formula: volume = 0.52ab2 (“a” indicates the long diameter and “b” is the short diameter). Once tumors reached between 120 and 300 mm^3^, mice were randomized according to tumor volume into groups of n = 7–8 mice per treatment. Drugs were administered once daily by oral gavage at 1mg/kg. Trametinib (GSK1120212) was dissolved in 0.5% hydroxypropylmethylcellulose (Sigma-Aldrich, St. Louis, MO, USA) and 0.2% Tween-80 pH 8.0.

### 4.11. RNA Preparation and Data Analysis for RNA-Seqs

RNA samples were extracted from cells treated by dimethylsulfoxide (DMSO) or 100 nM trametinib respectively. In each group, there were three parallels. All samples were saved in TRIzol reagent (Invitrogen). The following RNA library construction and sequencing were performed on a BGISEQ-500 platform in Beijing Genomic Institution (BGI, Shenzhen, China).

### 4.12. Statistical Methods

The data was represented as means ± standard errors of the mean (SEMs). The distribution types of the data were checked with the Kolmogorov–Smirnov normality test. For normal distribution, a two-tailed, unpaired student’s t-test was applied with GraphPad Prism 8 software. The Mann–Whitney test was performed for abnormal distribution to compare the statistical significance between the two groups. *p* values < 0.05 were considered as significant difference. Survival curves were constructed with the Kaplan–Meier method, and the differences between groups were assessed with the log-rank (Mantel-Cox) test.

## 5. Conclusions

In our study, we observed that a subtype of *KRAS*-mutant pancreatic cancer cells with higher expression of TAP1 were more resistant to MEKi. Suppression of TAP1 sensitized the resistant PDAC cells to MEKi whereas overexpression of TAP1 in sensitive PDAC cells conferred resistance to MEKi. Mechanistically, TAP1 (also named ABCB2) expression increased efflux of MEKi out of PDAC cells, leading to reduced concentration of MEKi in cells and development of MEKi resistance. Our results provide the potential ability of TAP1 in predicting the sensitivity of PDAC to MEK inhibition. It is suggested that TAP1 could act as a promising therapeutic target for improving the efficacy of MEKi in the treatment of MEKi-resistant PDAC.

## Data Availability

All data generated or analyzed during this study are included in this published article and its Supplementary Material.

## Supplementary Materials

The following supporting information can be downloaded at: https://www.mdpi.com/article/10.3390/ijms23137168/s1.

## Abbreviations

ABC	ATP-Binding Cassette
ERK	Extracellular-Signal Regulated Kinase
GEPIA	Gene Expression Profiling Interactive Analysis
GSEA	Gene Set Enrichment Analysis
IC50	Half-Maximal Inhibitory Concentration
KRAS	Kirsten Ras Oncogene Homolog from Ras Family
LC-MS/MS	Liquid Chromatograph-Tandem Mass Spectrometer
MEK	Mitogen-Activated Protein Kinase
MEKi	MEK Inhibitor
MRM	Multiple Reaction Monitoring
PDAC	Pancreatic Ductal Adenocarcinoma
TAP1	Transporter Associated with Antigen Processing 1
TCGA	The Cancer Genome Atlas
TMEM163	Transmembrane Protein 163
