# Supplementary material for: Antigen Peptide Transporter 1 (TAP1) Promotes Resistance to MEK Inhibitors in Pancreatic Cancers
Source: Int J Mol Sci. 2022 Jun 28;23(13):7168. doi: 10.3390/ijms23137168 (PMC9266799; doi:10.3390/ijms23137168)
Supplement: Supplementary file 1 [file ijms-23-07168-s001.zip › ijms-1771276-SI.pdf]

**Supplementary materials**

**Western blot**

Cell lysate samples were collected from cells and subjected to SDS-PAGE, then transferred to polyvinylidene difluoride membrane (Millipore, Billerica, MA, USA). The membrane was blocked with 5% fat-free milk and incubated with specific primary antibodies overnights at 4°C. And this was followed by incubation with HRP-conjugated secondary antibodies for 1 h at room temperature. The expression levels of proteins were detected with an enhanced chemiluminescence system (Thermo Fisher Scientific, Waltham, USA).

**Immunohistochemical (IHC) assay**

The tumor tissues of mice were deparaffinized and treated with 3% hydrogen peroxide for 10 min. 10 mmol/l sodium citrate buffer was used to retrieval antigen by heating for 15 min. Then the tissues were blocked with 10% goat serum and were incubated with primary antibody (1:200) at 4°C overnight. Then, the slides were incubated with HRP-conjugated secondary antibody for 1 h at room temperature.

**Immunofluorescence (IF)**

To stain the TAP1 protein at cell surface membrane, cells cultured on 96-well plates were blocked with 10% goat serum and stained with anti-TAP1 antibody overnight at 4°C. Then cells were fixed in 4% formaldehyde for 5 min. Binding of corresponding fluorescent-conjugated secondary antibodies was carried out at room temperature in dark for 1 h. Cells were subsequently stained for nucleus with DAPI. Images were acquired with Opera Phenix™ High Content Analysis System (PerkinElmer, Waltham, USA).

## Supplementary figures

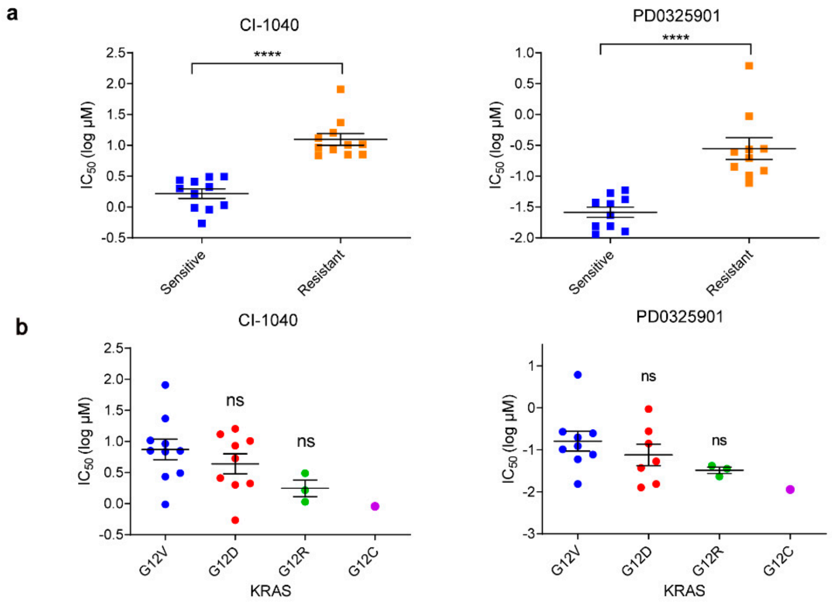


**Supplementary Figure S1. *KRAS*-driven pancreatic cancer cells have different sensitivities to MEK inhibitors**. (**a**) The response of *KRAS* mutation pancreatic cancer cell lines to CI-1040 and PD0315901. ****P < 0.0001. Data of logIC50 was from the Genomics of Drug Sensitivity in Cancer (GDSC) database. (**b**) IC50 values of cell lines with specific *KRAS* mutational subtypes were compared with cell lines with *KRAS* (G12V) to determine the P-value. Mann-Whitney test. ns, not significant.

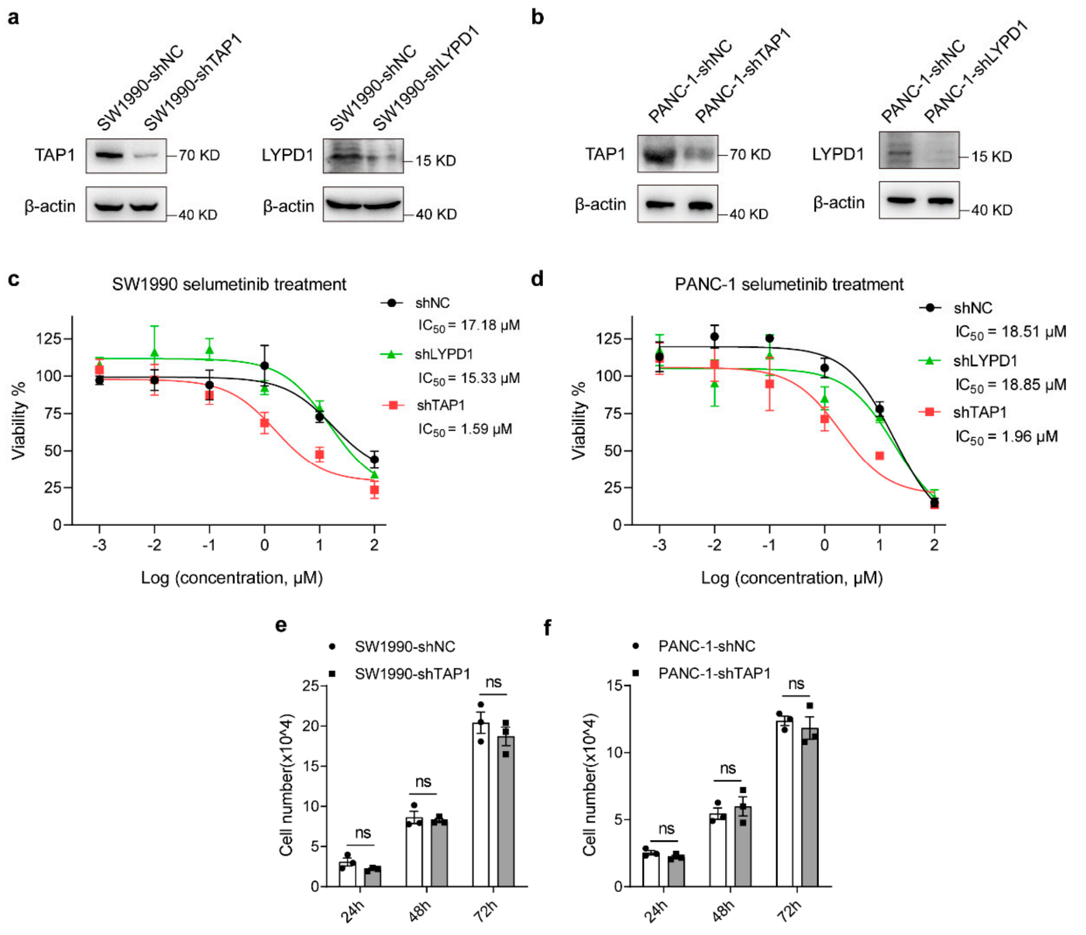


**Supplementary Figure S2. The sensitivity of MEKi-resistant cells to selumetinib is increased by TAP1 knockdown**. (**a, b**) Western blot of the expression level of TAP1 in SW1990 and PANC-1 cells with TAP1 shRNA or LYPD1 shRNA. Nontargeting shRNA (NC) was used as control. (**c, d**) Selumetinib sensitivities for SW1990 and PANC-1 cells with shTAP1, shLYPD1 and shNC were determined by CellTiter-Lumi™ assays. (**e, f**) The cell numbers of each cell lines were counting by Countstar software. ns, not significant.

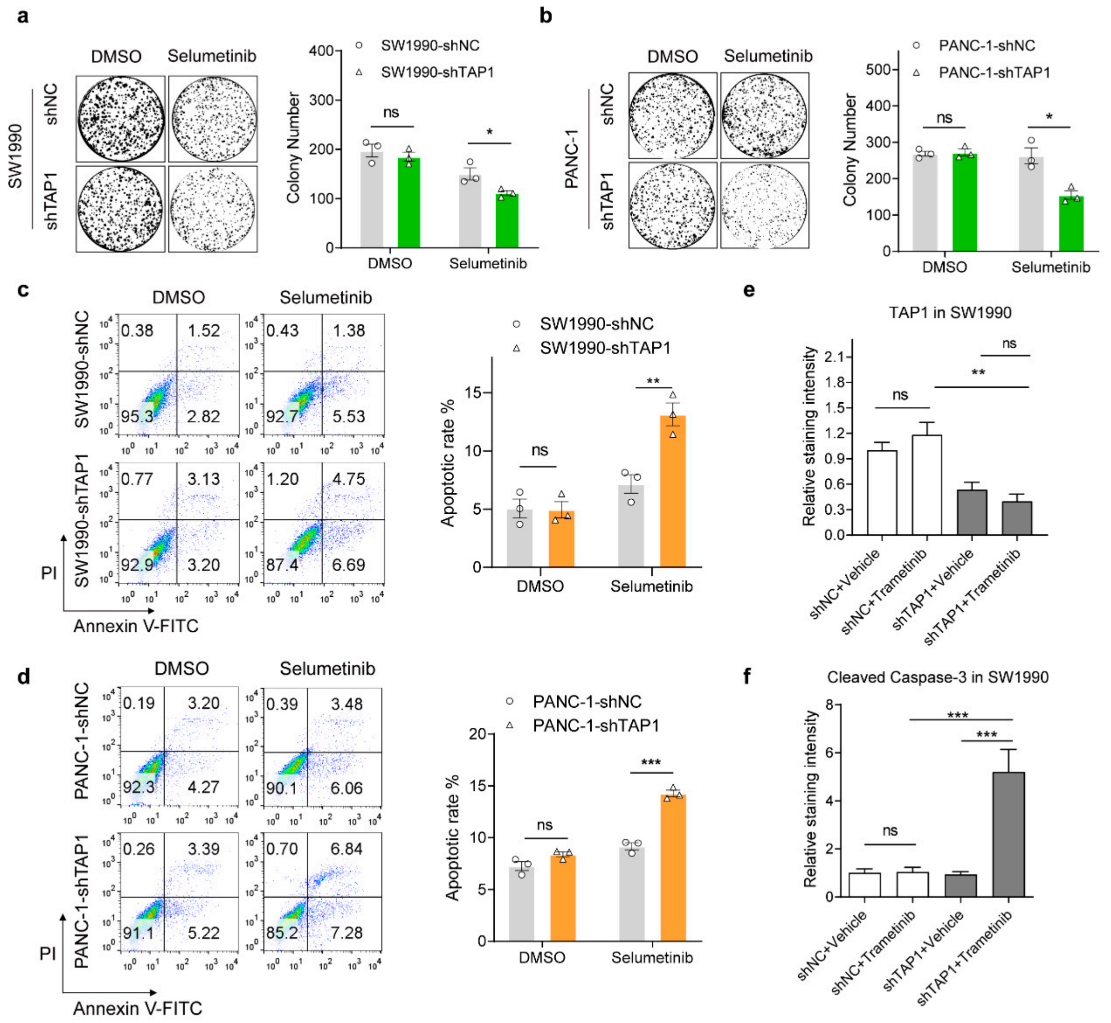


**Supplementary Figure S3. Suppression of TAP1 enhances the proliferation inhibitory and pro-apoptotic effect induced by selumetinib**. (**a, b**) Representative wells of the colony formation assay upon knockdown of TAP1 or NC in SW1990 and PANC-1 cells after selumetinib treatment for 12 days. Quantification of colony number was shown at the right. (**c, d**) Representative FCM images of shTAP1 or shNC in SW1990 and PANC-1 cells were treated by selumetinib for 72h. The quantitative data were showed at the right. (**e, f**) The relative staining intensity of TAP1 and cleaved caspase-3 in tumor tissues from the indicated groups of mice in figure 3i. Data were represented as mean ± SEM; **P < 0.01; ***P < 0.001; ns, not significant. Two-tailed t test.

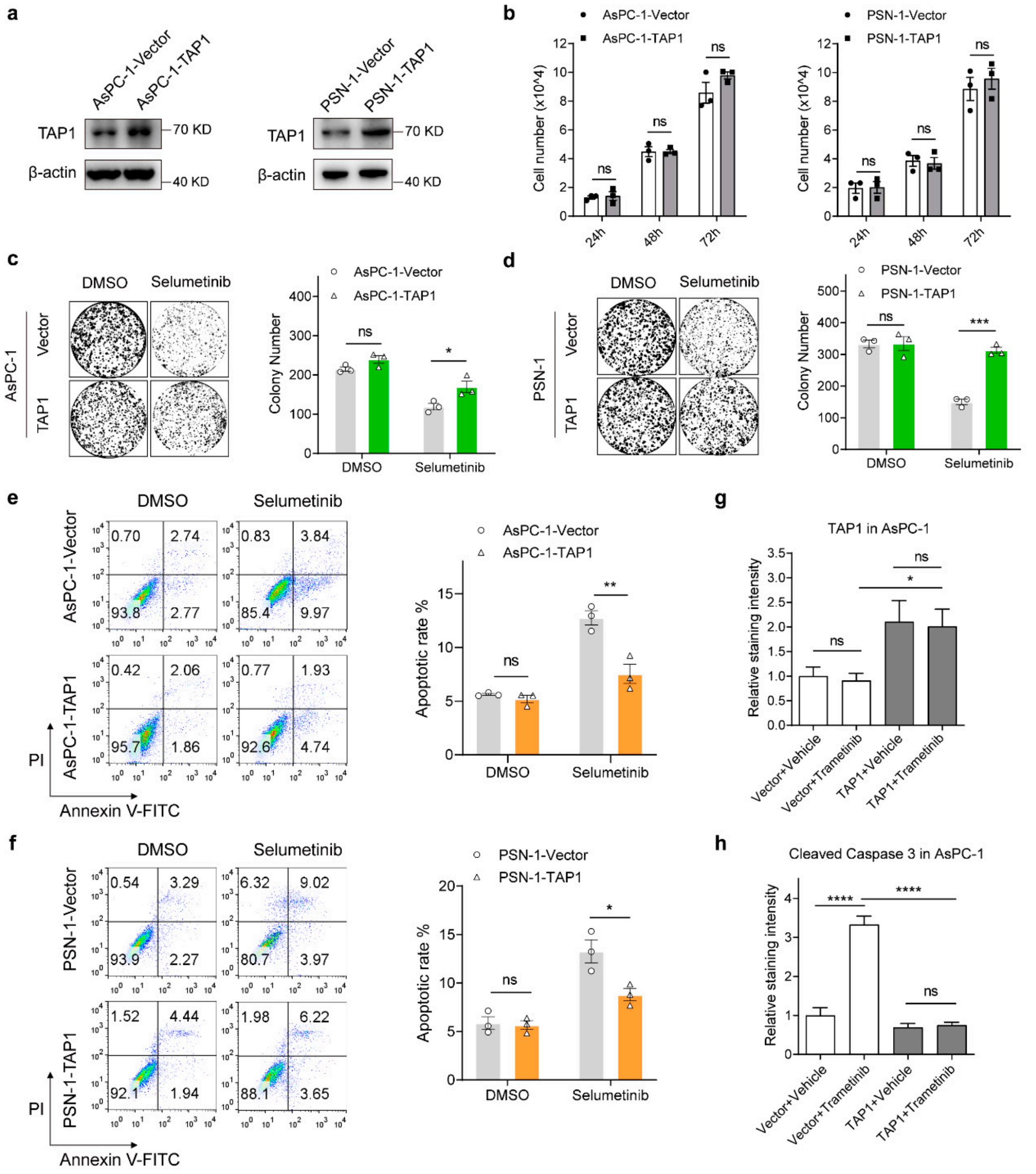


**Supplementary Figure S4. Overexpression of TAP1 increases the resistance of MEKi-sensitive cells to selumetinib treatment**. (**a**) Expression of TAP1 in TAP1-overexpression AsPC-1 and PSN-1 cells were examined by western blotting, compared to their repective vector control cells. (**b**) The cell numbers of each cell lines were counting by Countstar software. (**c, d**) Representative wells of the colony formation assay in TAP1-overexpressing AsPC-1 and PSN-1 cells under selumetinib treatment for 12 days. AsPC-1-Vector and PSN-1-Vector cells were as control. Quantification of colony number was shown at the right. (**e, f**) Representative FCM images of TAP1-ovexpessing AsPC-1 cells after treatment of selumetinib for 72 h. TAP1-Vector and PSN-1-Vector cells were as control. The quantitative data were showed at the right. (**g, h**) The relative staining intensity of TAP1 and cleaved caspase-3 in tumor tissues from the indicated groups of mice in figure 4k. Data were represented as mean ± SEM; *P < 0.05; ***P < 0.001; ****P < 0.0001; ns, not significant. Two-tailed t test.

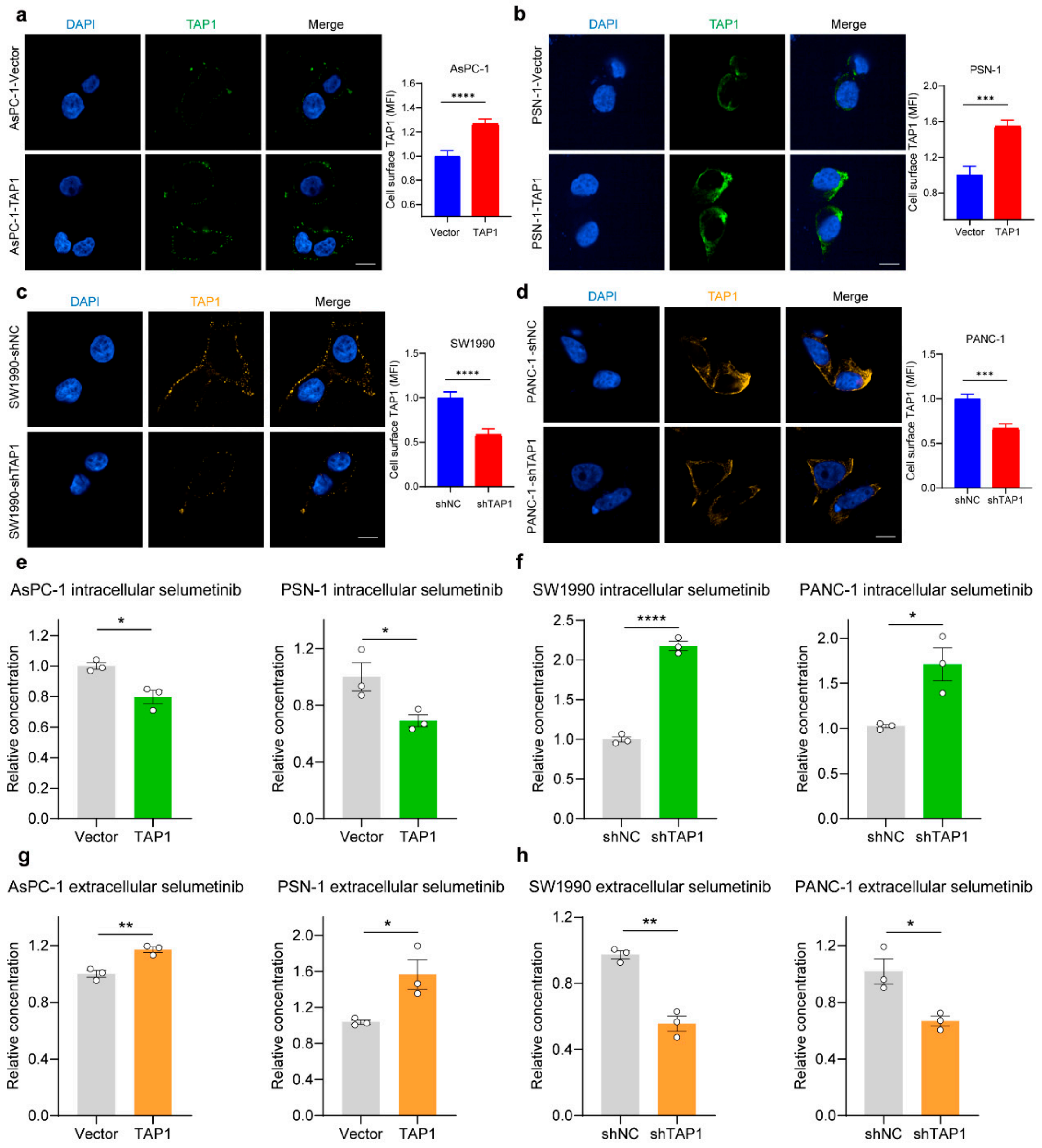


**Supplementary Figure S5. TAP1 overexpression increased the cell surface TAP1 expression and drug efflux in PDAC cells.** (**a, b**) Confocal microscopy showed the cell surface expression of TAP1 in AsPC-1 and PSN-1 cells with TAP1 overexpression and vector control cells. TAP1, green; DAPI, blue. Scale bars, 10 µm. Quantification data of was shown at the right. MFI, mean fluorescence intensity. (**c, d**) Cell surface expression of TAP1 in SW1990 and PANC-1 cells with TAP1 knockdown and control cells (shNC) were visualized by confocal microscope. TAP1, yellow; DAPI, blue. Scale bars, 10 µm. Quantification data of MFI was shown at the right. (**e, f**) Intracellular drug accumulation after incubation with 2 µM selumetinib in TAP1-overexpressing and TAP1-knockdown cells were determined by LC-MS/MS, compared to their respective control cells. (**g, h**) Measurement of extracellular selumetinib in the cell culture medium of TAP1-overexpressing and TAP1-knockdown cells by LC-MS/MS, compared to their respective control cells. Data were represented as mean ± SEM; *P < 0.05; **P < 0.01; ***P < 0.001; ****P < 0.0001; two-tailed t test.

**Supplementary tables**

**Table S1. Pancreatic cancer cells used for analyzed**

| Cell line | TCGA classification | Tissue | G12 |
|---|---|---|---|
| AsPC-1 | PAAD | pancreas | D |
| SUIT-2 | PAAD | pancreas | D |
| PSN1 | PAAD | pancreas | R |
| PA-TU-8902 | PAAD | pancreas | V |
| KP-2 | PAAD | pancreas | R |
| PANC-04-03 | PAAD | pancreas | D |
| PANC-03-27 | PAAD | pancreas | V |
| Capan-2 | PAAD | pancreas | V |
| PANC-10-05 | PAAD | pancreas | D |
| MZ1-PC | PAAD | pancreas | V |
| CFPAC-1 | PAAD | pancreas | V |
| DAN-G | PAAD | pancreas | V |
| MIA-PaCa-2 | PAAD | pancreas | C |
| KP-1N | PAAD | pancreas | D |
| HuP-T4 | PAAD | pancreas | V |
| YAPC | PAAD | pancreas | V |
| PANC-02-03 | PAAD | pancreas | D |
| HuP-T3 | PAAD | pancreas | R |
| CAPAN-1 | PAAD | pancreas | V |
| PL4 | PAAD | pancreas | D |
| SW1990 | PAAD | pancreas | D |
| KP-4 | PAAD | pancreas | D |
| SU8686 | PAAD | pancreas | D |
| PANC-08-13 | PAAD | pancreas | D |
| PA-TU-8988T | PAAD | pancreas | V |
| HPAF-II | PAAD | pancreas | D |

**Table S2. Primers used for qPCR**

| Primers used for qPCR | | |
|---|---|---|
| Gene | | Sequence |
| β-actin | F 5'-3' | ACTCTTCCAGCCTTCCTTCC |
| | R 5'-3' | CGTCATACTCCTGCTTGCTG |
| IFI44L | F 5'-3' | ACAGAGCCAAATGATTCCCTATG |
| | R 5'-3' | TCGATAAACGACACACCAGTTG |
| KRT17 | F 5'-3' | GGTGGGTGGTGAGATCAATGT |
| | R 5'-3' | CGCGGTTCAGTTCCTCTGTC |
| IFI27 | F 5'-3' | TGCTCTCACCTCATCAGCAGT |
| | R 5'-3' | CACAACTCCTCCAATCACAACT |
| IL8 | F 5'-3' | TTTTGCCAAGGAGTGCTAAAGA |
| | R 5'-3' | AACCCTCTGCACCCAGTTTTC |
| RBP1 | F 5'-3' | TCCAGTCACTCCCCGAAATG |
| | R 5'-3' | AGGTACTCCTCGAAATTCTCGTT |
| AADAT | F 5'-3' | AATTACGCACGGTTCATCACG |
| | R 5'-3' | TCCTCTGCTCAATATGTCAGTCA |
| TAP1 | F 5'-3' | TGCCCCGCATATTCTCCCT |
| | R 5'-3' | CACCTGCGTTTTCGCTCTTG |
| SLFN5 | F 5'-3' | GAGTGTGTTGTAGATGCAGGAA |
| | R 5'-3' | ACTGCTCGCAGGATGATTTCA |
| TMEM163 | F 5'-3' | CCGCCTGAAACCTCACGAA |
| | R 5'-3' | TCATAACGGAGACAGTAAAGGCA |
| INHBB | F 5'-3' | GTGAAGCGGCACATCTTGAG |
| | R 5'-3' | GCGAAGCTGATGATTTCGGAAAC |
| SCG5 | F 5'-3' | ATGCTATCTGGCCTACTGTTTTG |
| | R 5'-3' | GGCCCACAAGATTCATGGC |
| TCEA3 | F 5'-3' | CCCCAAAACACCTAGCAGC |
| | R 5'-3' | CTTCATGTCCGTGCTCTTGAG |
| AJUBA | F 5'-3' | ATGGGGAAGTCCTATCATCCAG |
| | R 5'-3' | TGGTAGTCGGTGACACAGTAT |
| MFI2 | F 5'-3' | ACCTCCTATTACGCCGTGG |
| | R 5'-3' | AGGGACTCAGAGTAACTGGTC |
| COL9A3 | F 5'-3' | GTGGATGGTCTGACTGGACG |
| | R 5'-3' | GGGCAGATACTTGGGCACTG |
| IL7R | F 5'-3' | TGTCGTCTATCGGGAAGGAG |
| | R 5'-3' | CGGTAAGCTACATCGTGCATTA |
| LYPD1 | F 5'-3' | GGCAACTTTTTGCGGATTGTT |
| | R 5'-3' | CGTTCACCGTGCAATTCACA |
| ID2 | F 5'-3' | GCTATACAACATGAACGACTGCT |
| | R 5'-3' | AATAGTGGGATGCGAGTCCAG |
| C10orf116 | F 5'-3' | CAGGAAACCATCGACAAGACTG |

| | | |
|---|---|---|
| | R 5'-3' | TCATTTCAGGAGGCCGAATTTTT |
| SLFN12 | F 5'-3' | TGAGTGACCTCGATGACTTAGAA |
| | R 5'-3' | ACACGGTTATCTTTCACATGCC |
| TMC8 | F 5'-3' | AGAACTACCCTCCCAACACG |
| | R 5'-3' | TTGTAGCCGTAGGACTCACAG |
| LCP1 | F 5'-3' | GATCAGTGTCCGATGAGGAAATG |
| | R 5'-3' | CCAGATCACCTGTAGCCATCA |
| GUCY1B3 | F 5'-3' | TGCTGGTGATCCGCAATTAC |
| | R 5'-3' | CCAGGACACGCAAGATTGTATC |
| ALDH1A1 | F 5'-3' | GCACGCCAGACTTACCTGTC |
| | R 5'-3' | CCTCCTCAGTTGCAGGATTAAAG |
| BMI1 | F 5'-3' | CCACCTGATGTGTGTGCTTTG |
| | R 5'-3' | TTCAGTAGTGGTCTGGTCTTGT |
| CXCR4 | F 5'-3' | ACGCCACCAACAGTCAGAG |
| | R 5'-3' | AGTCGGGAATAGTCAGCAGGA |
| EpCAM | F 5'-3' | TGATCCTGACTGCGATGAGAG |
| | R 5'-3' | CTTGTCTGTTCTTCTGACCCC |
| OCT4 | F 5'-3' | CTTGAATCCCGAATGGAAAGGG |
| | R 5'-3' | GTGTATATCCCAGGGTGATCCTC |
| c-Myc | F 5'-3' | CGACTCTGAGGAGGAACAAG |
| | R 5'-3' | GTGATCCAGACTCTGACCTT |
| NESTIN | F 5'-3' | GGCGCACCTCAAGATGTCC |
| | R 5'-3' | CTTGGGGTCCTGAAAGCT |
